# Properties of non-coding mutation hotspots as urinary biomarkers for bladder cancer detection

**DOI:** 10.1038/s41598-023-27675-4

**Published:** 2023-01-19

**Authors:** L. Baxter, N. S. Gordon, S. Ott, J. Wang, P. Patel, A. Goel, K. Piechocki, L. Silcock, C. Sale, M. P. Zeegers, K. K. Cheng, N. D. James, The BladderPath trial management group, A. Knight, J. Gallagher, S. Magwaro, A. Hughes, A. Pope, N. Tunariu, H. Doyle, W. Liu, H. Mintz, V. Nanton, K. James, S. Hafeez, J. W. F. Catto, K. Jefferson, S. J. Pirrie, R. T. Bryan, D. G. Ward

**Affiliations:** 1 Bioinformatics Research Technology Platform, University of Warwick, Coventry, UK; 2 Bladder Cancer Research Centre, Institute of Cancer and Genomic Sciences, College of Medical and Dental Sciences, University of Birmingham, Birmingham, B15 2TT UK; 3 University of Warwick Medical School, University of Warwick, Coventry, UK; 4 University Hospitals Birmingham NHS Foundation Trust, Birmingham, UK; 5 Nonacus Ltd, Birmingham Research Park, Birmingham, UK; 6 Department of Epidemiology, Care and Public Health Research Institute, School of Nutrition and Translational Research in Metabolism, Maastricht University, Maastricht, The Netherlands; 7 Institute of Applied Health Research, College of Medical and Dental Sciences, University of Birmingham, Birmingham, UK; 8 Institute of Cancer Research, London, UK; 9 Patient Advocate, London, UK; 10 Cancer Research UK Clinical Trial Unit, University of Birmingham, Birmingham, UK; 11 The Royal Marsden, London, UK; 12 University of Sheffield Medical School, Sheffield, UK; 13 University Hospitals Coventry and Warwickshire NHS Trust, Coventry, UK

**Keywords:** Diagnostic markers, Cancer

## Abstract

Mutations at specific hotspots in non-coding regions of *ADGRG6, PLEKHS1, WDR74, TBC1D12* and *LEPROTL1* frequently occur in bladder cancer (BC). These mutations could function as biomarkers for the non-invasive detection of BC but this remains largely unexplored. Massively-parallel sequencing of non-coding hotspots was applied to 884 urine cell pellet DNAs: 591 from haematuria clinic patients (165 BCs, 426 non-BCs) and 293 from non-muscle invasive BC surveillance patients (29 with recurrence). Urine samples from 142 non-BC haematuria clinic patients were used to optimise variant calling. Non-coding mutations are readily detectable in the urine of BC patients and undetectable, or present at much lower frequencies, in the absence of BC. The mutations can be used to detect incident BC with 66% sensitivity (95% CI 58–75) at 92% specificity (95% CI 88–95) and recurrent disease with 55% sensitivity (95% CI 36–74) at 85% specificity (95% CI 80–89%) using a 2% variant allele frequency threshold. In the NMIBC surveillance setting, the detection of non-coding mutations in urine in the absence of clinically detectable disease was associated with an increased relative risk of future recurrence (RR = 4.62 (95% CI 3.75–5.48)). As urinary biomarkers, non-coding hotspot mutations behave similarly to driver mutations in BC-associated genes and could be included in biomarker panels for BC detection.

## Introduction

*TERT* promoter mutations are present in 70–80% of BCs and have been extensively studied as urinary biomarkers for BC^1,2^. Further recurrent mutations hotspots in non-coding regions of genes have since been discovered in other cancers^3,4^. In a whole genome sequencing study of 65 BCs Wu et al.^5^ identified, in addition to the *TERT* promoter, the most frequent non-coding mutation hotspots as being present in *ADGRG6, PLEKHS1, TBC1D12, WDR74* and *LEPROTL1*. We subsequently confirmed these findings and reported the frequencies of the mutations in 302 incident BCs, finding mutations in at least one hotspot in 74% of tumours^6^. The mutation hotspots are all within palindromic sequences that are prone to APOBEC mutagenesis and it is likely that they are passenger mutations resulting from APOBEC activity rather than BC-driver mutations^7–9^, perhaps with the exception of the chr10: 96162368 C/T mutation in *TBC1D12*^3,7,10^. As such, the mutations cannot be considered as useful urinary biomarkers for BC until the distribution of the mutations in the urine of patients with and without BC have been defined. Two studies have examined *PLEKHS1* and *TBC1D12* mutations in the urine of BC patients and non-cancer control subjects; however, both had limited samples sizes and used healthy controls^11,12^. The variant allele frequencies of the non-coding hotspots in urine have also been shown to decrease following chemoradiotherapy in BC patients and following upper-tract tumour resection^13,14^.

Multiple studies have shown that coding mutations in BC-associated genes and the *TERT* promoter can be used for the non-invasive detection of BC^11,15,16^. We hypothesized that non-coding mutation hotspots may also prove useful as urinary biomarkers for BC detection even if they represent APOBEC mutagenesis rather than carcinogenesis directly, since, in the absence of a tumour, there should be limited clonal expansion of mutated cells and hence only low levels of mutations in the urine^17,18^. To test this hypothesis we used error-suppressed ultra-deep sequencing to study the mutations in 884 urine cell pellet DNAs (cpDNAs) from patients attending “haematuria” and “surveillance” clinics, including nearly 700 real-world controls.

## Methods

### Patients and samples

The data reported here were generated in parallel with the data recently reported in reference^16^. Additionally, non-coding mutation hotspot data were previously reported for tumour tissue from 23 of the patients^6^. Patient demographics are shown in in Table 1. The presence/absence of BC on the date of urine collection was determined by flexible cystoscopy and TURBT pathology. All included BCs were purely or predominantly urothelial carcinomas and were classified according to grade (WHO 1973^19^) and stage (UICC). All patients provided informed consent in writing. Results were not reported back to patients and did not influence patient care. Sample collection and experimental procedures were in accordance with the ethical approvals listed below and the Declaration of Helsinki. Sample size calculations were based on 90% target sensitivity and specificity with a lower 95% confidence interval (CI) limit close to 85%; 150 patients per group would yield 95% CIs of 83.8–94.1%.

**Table 1:** Patient characteristics.

Cohort	Disease status	No	M/F	Age (years)	Stage pTis/Ta/T1/T2 + /nr	Grade Grade 1/2/3/nr
Haematuria Clinic	Non-BC (training)	142	73/69	59.5	na	na
	Non-BC (test)	284	145/139	58.5	na	na
	BC	165	132/33	72.5	3/88/34/37/3	24/53/77/11
NMIBC Surveillance	Non-BC	264	175/89	74	na	na
	BC	29	18/11	71	0/17/4/2/6	4/8/11/6

M = male, F = female, age = median age, stage/grade: nr = not recorded.

The Haematuria Clinic cohort included 494 samples prospectively collected for this study from haematuria clinic patients, initially from The Queen Elizabeth Hospital, Birmingham, UK (North West—Haydock Research Ethics Committee approval: 15/NW/0079), and subsequently from other UK urology units participating in the BladderPath study (London Bridge Research Ethics Committee approval: 17/LO/1819)^20^. Specimens were collected on the day of clinic attendance, before being transferred/posted to the Human Biomaterials Resource Centre (HBRC) at the University of Birmingham for centrifuging and freezing. The non-BCs were determined to be ‘normal’ or with diagnoses including calculi, benign prostatic hyperplasia, cystitis, inflammation, urinary tract infection and kidney cancer. The haematuria cohort also included 97 urine samples from the Bladder Cancer Prognosis Programme (BCPP) prospectively collected for biomarker research at 10 West Midlands (UK) hospitals between 2005 and 2011 (East Midlands—Derby Research Ethics Committee approval: 06/MRE04/65). Patients without a prior history of BC were recruited on the basis of cystoscopic suspicion of primary BC; specimens were collected prior to treatment and were immediately centrifuged and frozen^21^.

The non-muscle invasive BC (NMIBC) Surveillance cohort samples were collected from NMIBC patients attending cystoscopy surveillance clinics at The Queen Elizabeth Hospital, Birmingham, UK (ethics ref. 15/NW/0079). Specimens were collected on the day of clinic attendance, before being transferred to HBRC for processing and freezing.

DNA was extracted from cell pellets using the Quick-DNA Urine Kit (Zymo Research D3061) and quantitated using the high-sensitivity dsDNA Qubit kit (Thermofisher).

### Library preparation and sequencing

Libraries were prepared using the Nonacus Cell3 Target enrichment protocol according to the manufacturer’s instructions using 25 ng urine cpDNA. DNA was enzymatically sheared, end-repaired and A-tailed, and adapters (including UMIs) ligated to the fragments. Libraries were amplified and pooled in batches of 12 prior to overnight hybridisation with biotinylated probes and subsequent capture and final amplification of the NGS libraries. The probes targeted hotspots or regions of 28 genes including the 5 reported here^16^. A full protocol can be found at www.nonacus.com. All libraries were 2 × 150 bp sequenced on a Novaseq (Illumina).

### Bioinformatics

Sequencing data were de-multiplexed and aligned to hg19 using bwa (version 0.7.15-r1140). Consensus reads were built using fgbio (version 1.1.0) requiring ≥ 3 reads to produce a consensus as described previously^16^ and re-aligned to the reference. Average raw and consensus read depths were 35,400 × and 2400 × respectively. Base calls with quality ≥ 30 were extracted using bam-readcount, and used to calculate variant allele frequencies (VAFs) at the coordinates of the 19 non-coding hotspots in the 5 genes^6^. A variant calling strategy was developed based on the maximum VAFs observed in a randomly-selected training set of BC-negative haematuria patients.

## Results

### Variant calling optimisation

**Figure 1:**
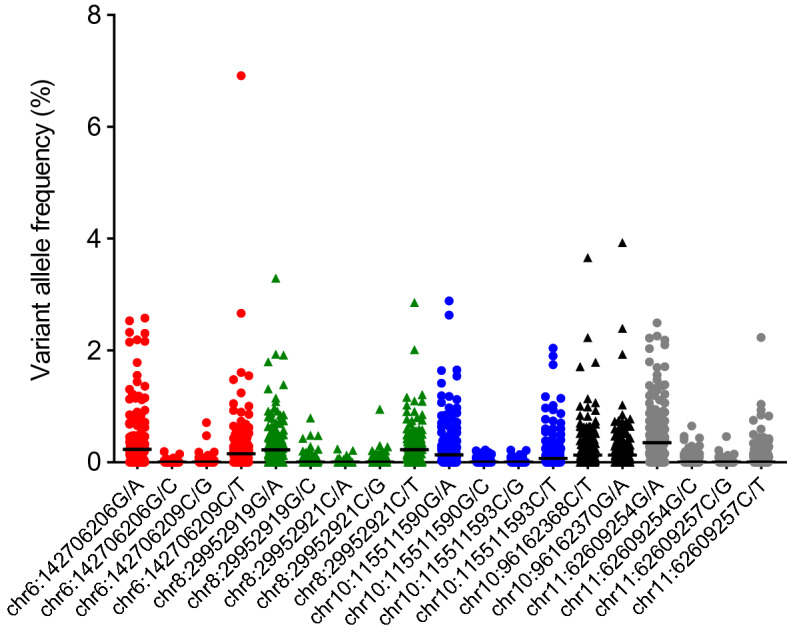
Variant allele frequencies at non-coding mutation hotspots in urine cpDNA from haematuria clinic non-BC patients. Red circles = *ADGRG6*, green triangles = *LEPROTL1*, blue circles = *PLEKHS1*, black triangles = *TBC1D12*, grey circles = *WDR74*. Data are shown for the training set (n = 142). Horizontal bars represent median values.

One-third of the haematuria clinic non-BC samples were used as a training set for determining thresholds for variant calling. The maximum VAFs observed were below 3% in 140 out of 142 non-BC patients but were substantially higher than the intrinsic error rate of the method, suggesting that the mutations are present at low levels in the urine of some non-BC haematuria patients (Fig. 1). The median maximum VAF at any hotspot in all 142 samples was 0.65% with higher medians observed for the mutations that are most frequently observed in BCs. The median maximum VAF at any hotspot in 27 germline DNA samples (blood) samples was 0.28%, and this was largely attributable to the *WDR*74 mutation chr11:62609254G/A; excluding this mutation decreased the median maximum VAF to 0.09% across all of the other hotspots and left no mutations at > 0.5% VAF in germline DNA (data not shown). If we were to develop a urine test for BC and define detection of a single mutation as a positive result, then we need to set a threshold for mutation detection. Accordingly, on these data, if we set the threshold at 1, 1.5 or 2% for the maximum VAF in each sample, we obtain specificities of 68, 86 and 90% respectively. Reasoning that if the non-coding mutations are to be combined into a biomarker panel in the future, then not compromising the specificity of the overall panel would be important, we decided to use 2% VAF for further testing of biomarker performance. Analysis of a quality control sample (pooled tumour and germline DNA) using the 2% threshold identified mutations at 5% VAF in 17 out of 17 technical replicates and mutations at 2.5% VAF in 14/17 (82%) replicates indicating highly reproducible mutation detection.

### Sensitivity and specificity for incident disease detection

**Figure 2:**
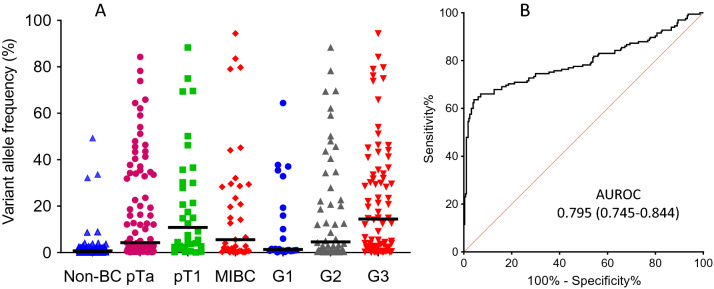
Hotspot mutations  in urine cpDNAs from incident BC patients. (**A**) Shows the highest VAFs in cpDNAs from the testing set non-BCs (n = 284) and 165 BCs by stage and grade. (**B**) Shows the ROC curve for incident BC detection obtained by varying the VAF used for variant calling.

We used the 2% VAF strategy defined on the training set to call variants in cpDNAs from the remainder of the haematuria clinic cohort. Of 165 BC cases, 109 tested positive (66% sensitivity (95% CI 58–73%)) and of 284 test set non-BCs, 261 tested negative (92% specificity (95% CI 88–95%)). Mutations were detected far more commonly in cpDNA from patients with all stages and grades of BC compared with non-BC patients (p < 0.001) (Fig. 2a). The median maximum VAF in the incident BCs was 5.97%, versus 0.73% in non-BC patients (p < 0.001). The area under an ROC curve generated by varying the VAF threshold for variant calling was 0.795 (95% CI 0.745–0.844) (Fig. 2b). Mutation frequencies for individual genes by stage and grade are shown in Table 2.

**Table 2:** Non-coding mutation hotspots in urine cpDNAs across stages and grades of incident BC.

	G1pTa *n* = *23*	G2pTa *n* = *42*	G3pTa *n* = *15*	G2pT1 *n* = *8*	G3pT1 *n* = *24*	MIBC *n* = *36*	Other BC *n* = *17*	All BCs *n* = *165*	Non-BCs *n* = *284*
*ADGRG6*	7	19	9	5	15	13	6	74	14
*PLEKHS1*	5	11	9	5	11	9	9	58	6
*WDR74*	5	11	7	7	11	9	6	53	7
*TBC1D12*	4	11	10	3	2	8	5	44	5
*LEPROTL1*	1	12	8	4	7	5	3	40	4
Any	9	25	15	6	21	22	11	109	23
Sensitivity	39	60	100	75	88	61	65	66	na
Specificity	Na	na	na	na	na	na	na	na	92

The table shows the number of cpDNAs in which a mutation in each gene was detected with > 2% VAF. Other BC = stage or grade unrecorded or CIS (n = 3) or G1pT1 (n = 1). Any = mutation(s) detected in ≥ 1 gene. The non-BCs used in the training set have been excluded. Sensitivity and specificity are shown as percentages.

### Relationship between APOBEC mutagenesis and age in non-BC patients

**Figure 3:**
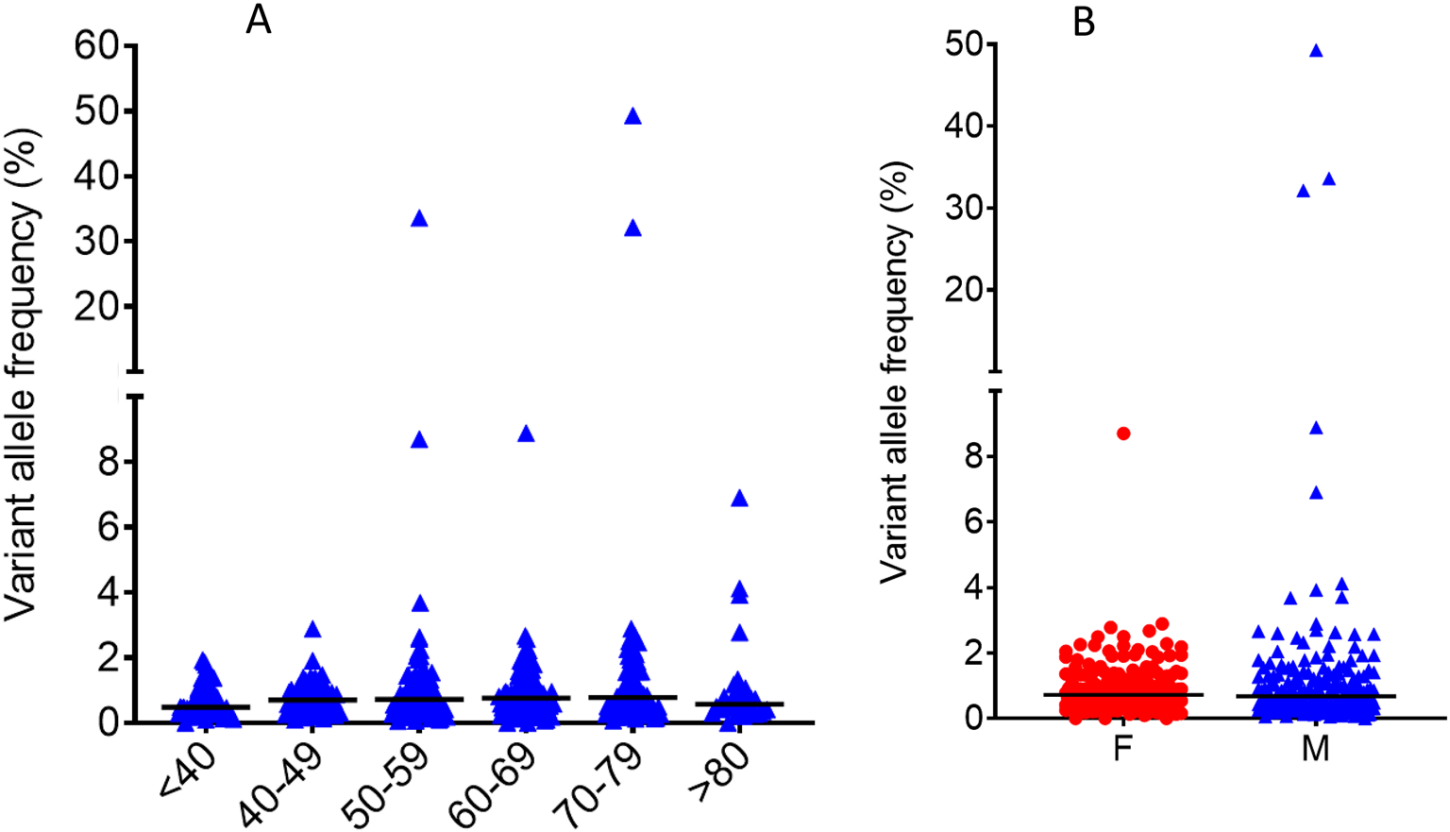
Effects of age (**A**) and gender (**B**) on non-coding mutations in non-BC haematuria clinic patients. The data are non-coding hotspot maximum VAFs in individual cpDNAs (all non-BC patients). Horizontal bars represent median values.

We used the data from all of the BC-free haematuria clinic patients to investigate how the frequencies of mutations at the non-coding hotspots depend on sex and patient age in the absence of BC (Fig. 3). Overall, the mutation levels (maximum VAF in each sample) do not show a strong tendency to increase with age; however, the false-positive rate was < 1% in patients below 50 years of age, 9% in patients aged 50–65 and 14% in patients aged 65 or over, suggesting an increased chance of clonal expansion of cells with APOBEC mutations with age even in the absence of clinically-detectable BC. VAFs did not differ significantly between male and female patients.

### Sensitivity and specificity for detecting recurrent disease

**Figure 4:**
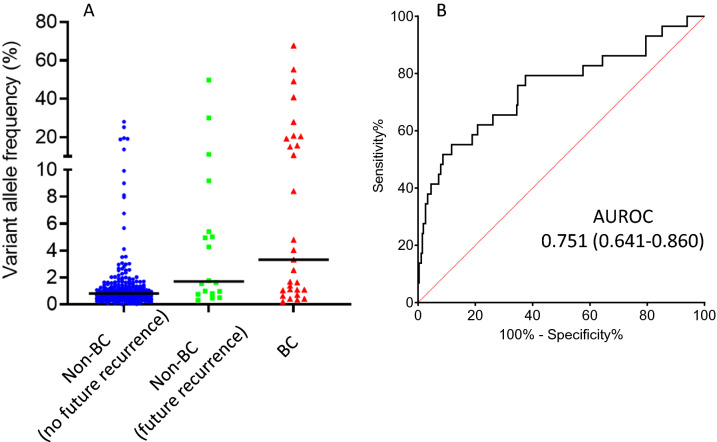
Hotspot mutations in urine cpDNAs from NMIBC surveillance patients. (**A**) Shows VAFs in surveillance clinic cpDNAs. Horizontal bars represent median values. (**B**) Shows the ROC curve for recurrent BC detection obtained by varying the VAF used for variant calling.

We analysed the urines from 256 NMIBC surveillance patients; 37 of these patients underwent testing at two separate surveillance cystoscopy episodes, resulting in a total of 293 cpDNAs for analysis. On the day of urine collection, 29 and 264 cpDNAs were from patients with or without cystoscopy-detectable recurrences, respectively. The median maximum VAF was significantly higher in BC recurrence cpDNAs than non-recurrent cpDNAs (3.32% v 0.81%, p < 0.001) (Fig. 4). Applying the same variant calling strategy used with the haematuria clinic samples, we obtained 55% sensitivity (95% CI 36–74%) at 85% specificity (95% CI 80–89%) in the surveillance setting. Varying the VAF threshold generated the ROC plot shown in Fig. 4b with an area of 0.751 (95% CI 0.641–0.860).

Of the 264 cpDNAs from patients cystoscopically negative for BC on the day of urine collection, 18 were from patients diagnosed with recurrence at their next cystoscopy 2–24 months later. In the pre-recurrence cpDNAs the VAFs were significantly higher than in the 246 cpDNAs from patients without subsequent recurrence (1.69% vs 0.81%, p = 0.016) (Fig. 3); 8 of 18 cpDNAs (44%) from patients with future recurrence tested positive, compared with 31 of 246 cpDNAs (13%) from patients without subsequent recurrence. The presence of non-coding mutations in urine in the absence of clinically detectable disease was associated with an increased risk of future recurrence (RR = 4.62 (95%CI 3.75–5.48)).

## Discussion

Mutations frequently occur at non-coding hotspots in *ADGRG6, PLEKHS1, TBC1D12, WDR74* and *LEPROTL1* in BC, whereas mutations in the coding regions of these genes are rare. *LEPROTL1* is listed in the COSMIC Cancer Gene Census as a tumour suppressor gene and is deleted in 5% of MIBCs; however, this does not appear to impact prognosis^22^. The mRNAs of all 5 genes are expressed in bladder tumour tissues and high levels of *PLEKHS1* mRNA are a good prognostic indicator in MIBC^22^. We previously reported that non-coding mutations in *PLEKHS1* and *WDR74* are good prognostic indicators in a cohort of 302 BC patients, but that the non-coding mutations do not appear to alter the expression or splicing of any of the 5 genes^6^. Thus, although a role for any of these genes in urothelial carcinogenesis remains to be proven, due to their high mutation frequencies they could be useful as urinary biomarkers for BC detection and/or surveillance. They would need to be used either with a tumour-informed approach or in combination with additional biomarkers as they are only present in 74% of BCs^6^. Notwithstanding, we have presented the first large scale measurement of these mutations in the urine of BC patients and non-BC patients. Mutations are detected in urine above background levels in 66% of incident BC patients, closely mirroring the 74% reported in BC tissue^6^.

An interesting finding is that mutations at the non-coding hotspots are found at low, but measurable, levels in many haematuria patients in the absence of clinically detectable BC. This would suggest that APOBEC mutagenesis is a common feature of the urothelium that only rarely leads to bladder cancer. Recent multi-region sequencing studies have identified mutations in macroscopically normal urothelium^17,18^. Although both studies used gene-panel or exome sequencing and so do not provide information directly on the non-coding hotspots, Lawson et al.^17^ identified APOBEC signatures in 22% of urothelial microbiopsies from non-BC patients. Our data are consistent with there being multiple individual cells or small patches of cells with non-coding mutations in apparently normal urothelium but which only comprise a small fraction of the urothelium. Detection of these mutations at VAFs > 2% in urine cell pellets only commonly occurs with the large-scale clonal expansion of carcinogenesis.

The non-coding hotspot mutation data described here were acquired in parallel with mutations in the 23-gene panel previously described^16^. The 23-gene panel was able to detect incident BC with a sensitivity of 87% at 85% specificity. The non-coding mutations achieved 66% sensitivity at 92% specificity and we investigated the combination of the two sets of data; however, including the non-coding mutations did not increase sensitivity (data not shown). As the 23-gene panel can theoretically detect 96% of all BCs^23^, it is perhaps not surprising that the non-coding mutations do not increase sensitivity: it is likely that very low levels of tumour DNA in some urine samples, rather than a lack of mutations in BCs, is the main cause of false negative results. Nonetheless, including the non-coding mutations increases the average number of mutations in each BC urine sample from 3 for the 23-gene panel to 5. This could potentially increase confidence in test results and aid in the development of a tumour-agnostic panel for plasma ctDNA analysis (where very low VAFs can make detection of individual mutations subject to stochastic variability)^24^. Alternatively, assays for the non-coding mutations could be used in tumour-informed liquid biopsy analyses for the majority of patients (rather than having to develop new patient-specific assays each time as required with rarer mutations).

## Conclusions

We have presented an evaluation of non-coding mutations as urinary biomarkers for BC. Although there is a low-level mutation background in the absence of BC, the mutations can be detected at VAFs > 2% in the cpDNA of two-thirds of incident BC patients and over half of recurrent NMIBCs. Our data suggest that the non-coding mutations could be incorporated into a focused mutation panel for BC detection.

## Data Availability

The data is available at the EGA European Genome-Phenome Archive: EGAS00001006349.

## References

[1] Allory Y (2014). Telomerase reverse transcriptase promoter mutations in bladder cancer: High frequency across stages, detection in urine, and lack of association with outcome. Eur. Urol..

[2] Hurst C, Platt F, Knowles M (2014). Comprehensive mutation analysis of the TERT promoter in bladder cancer and detection of mutations in voided urine. Eur. Urol..

[3] Rheinbay E (2017). Recurrent and functional regulatory mutations in breast cancer. Nature.

[4] Weinhold N (2014). Genome-wide analysis of noncoding regulatory mutations in cancer. Nat. Genet..

[5] Wu S (2019). Whole-genome sequencing identifies ADGRG6 enhancer mutations and FRS2 duplications as angiogenesis-related drivers in bladder cancer. Nat. Commun..

[6] Jeeta R (2019). Non-coding mutations in urothelial bladder cancer: Biological and clinical relevance and potential utility as biomarkers. Bladder Cancer.

[7] Buisson R (2019). Passenger hotspot mutations in cancer driven by APOBEC3A and mesoscale genomic features. Science.

[8] Vacher S (2020). Genomic instability signature of palindromic non-coding somatic mutations in bladder cancer. Cancers (Basel)..

[9] Shi M-J (2020). Identification of new driver and passenger mutations within APOBEC-induced hotspot mutations in bladder cancer. Genome Med..

[10] Yang A, Cross C, Townsend J (2020). Non-coding mutations in urothelial bladder cancer: Biological and clinical relevance and potential utility as biomarkers. Bladder Cancer.

[11] Dudley J (2019). Detection and surveillance of bladder cancer using urine tumor DNA. Cancer Discov..

[12] Chauhan P (2021). Urine tumor DNA detection of minimal residual disease in muscle-invasive bladder cancer treated with curative-intent radical cystectomy: A cohort study. PLoS Med..

[13] Gordon N (2022). Urine DNA for monitoring chemoradiotherapy response in muscle-invasive bladder cancer: A pilot study. BJU Int..

[14] Xing X (2021). Regulatory region mutations of TERT, PLEKHS1 and GPR126 genes as urinary biomarkers in upper tract urothelial carcinomas. J Cancer..

[15] Springer S (2018). Non-invasive detection of urothelial cancer through the analysis of driver gene mutations and aneuploidy. Elife.

[16] Ward D (2022). Highly sensitive and specific detection of bladder cancer via targeted ultra-deep sequencing of urinary DNA. Eur. Urol. Oncol..

[17] Lawson A (2020). Extensive heterogeneity in somatic mutation and selection in the human bladder. Science.

[18] Li R (2020). Macroscopic somatic clonal expansion in morphologically normal human urothelium. Science.

[19] Mostofi, F. *et al*. *Histological Typing of Urinary Bladder Tumours.* https://apps.who.int/iris/handle/10665/41533. (1973).

[20] Bryan R (2021). Comparing an imaging-guided pathway with the standard pathway for staging muscle-invasive bladder cancer: Preliminary data from the Bladderpath study. Eur. Urol..

[21] Zeegers M (2010). The West Midlands Bladder Cancer Prognosis Programme: Rationale and design. BJU Int..

[22] Robertson A (2017). Comprehensive molecular characterization of muscle-invasive bladder cancer. Cell.

[23] Ward D (2019). Targeted deep sequencing of urothelial bladder cancers and associated urinary DNA: A 23-gene panel with utility for non-invasive diagnosis and risk stratification. BJU Int..

[24] Deveson I (2021). Evaluating the analytical validity of circulating tumor DNA sequencing assays for precision oncology. Nat. Biotechnol..

